# The Past, Present, and Future Care for Refractory Chronic Cough in Children and Adults

**DOI:** 10.3390/jcm14186594

**Published:** 2025-09-18

**Authors:** Miles Weinberger, Ran D. Anbar, Dennis Buettner

**Affiliations:** 1 Rady Children’s Hospital, University of California San Diego, San Diego, CA 92123, USA; 2 Center Point Medicine, La Jolla, CA 92037, USA; 3 Habit Cough Association, Severna Park, MD 21146, USA; dennis.buettner@gmail.com

**Keywords:** behavioral therapy, clinical education, unexplained refractory chronic cough, habit cough, age-specific cough etiology, hypnosis, suggestion therapy, video modeling

## Abstract

Chronic cough without explanation or diagnosis has been described in medical books for over 300 years. Since 1977, some comorbidities, such as gastroesophageal reflux and post-nasal drip, have been attributed to be causes of otherwise unexplained chronic cough. Since 2005, publications have reported that unexplained chronic cough could be a distinct entity refractory to usual treatment. This was explained by dysregulation of cough centers involving the brainstem, subcortical, and cortical areas for which a neuromodulator would be essential for treatment. However, an 1886 publication described this disorder as a type of involuntary habit, and recommended treatment to break the habit. Supporting this alternative paradigm were studies that showed the urge to cough may have resulted from local airway inflammation with increased mucosal nerve density caused by the frequent daily coughing. The result was a vicious cycle where coughing caused the urge to cough, leading to repetitive daily coughing. Treatment has been demonstrated by a behavioral technique that breaks the habit. That behavioral technique, called suggestion therapy, has been highly successful in children and is now recognized as the standard of care for this disorder at pediatric referral centers. The proof of concept that suggestion therapy is effective in adults with refractory chronic cough has been demonstrated in selected adults but is not yet a common practice at specialty cough centers.

## 1. Introduction

Occasional acute cough is universal. Chronic cough occurs only in some. When present, it can be a miserable problem. It inhibits social activity, school attendance for children, and occupational activity for adults. Chronic cough can be associated with various clinical disorders including asthma [1], COPD [2], pulmonary fibrosis [3], inhibitors of angiotensin-converting enzyme [4], and occasionally anatomic abnormalities of the upper airway [5]. The frequency of chronic cough is reported to be as high as 10% of the adult population, but varies in different publications [6]. Consequently, physicians frequently encounter patients with chronic cough.

This review focuses on chronic cough that is refractory to usual guidelines for pharmacological treatment as outlined in a 2018 expert cough panel report [7]. Much of the current assessment and treatment of chronic cough has been based on medical experience over the past 50 years. To understand current common medical practice of refractory chronic cough, we review the beliefs and practice that have been the basis of the various published recommendations and guidelines [8,9,10]. We review the clinical experience of the past, current controversies of care, and the potential for future improvement in care.

## 2. The Past

Chronic cough unrelated to an etiology has been described in medical texts for over 300 years and has often been referred to as habitual cough [11,12]. Willis in 1685 described a woman with “…a violent dry Cough following her day and night, unless when she was fallen asleep.” [11]. This description is consistent with the current definition of the habit cough disorder [13]. Mercurius in 1694 described a “…habitual cough, which often continues after the cough caused by a cold is gone….” [12]. This is consistent with histories commonly obtained from parents of children and adults with refractory chronic cough. Charles Creighton in 1886 described, “…a habit cough—a reflex effect persisting after the cause is gone …. or an acquired habit….the treatment of it is to break the habit…” [14]. Dr. Creighton’s insightful comments are consistent with the subsequent description of a curative procedure for habit cough [15].

Despite these early descriptions, chronic cough as an entity did not appear in the medical lexicon until much later. Chronic cough was a rare to nonexistent subject in medical school, nor is chronic cough mentioned in the massive two volumes of the 2001 15th Edition of Harrison’s Principles of Internal Medicine. No diagnostic code for chronic cough was in ICD9 which was used from 1979 until the 2015 change to ICD10. Awareness of chronic cough reappeared, but as a symptom of some disorder such as asthma, post-nasal drip, and/or gastrointestinal reflex.

Richard Irwin was a major contributor to 20th century publications on chronic cough. In a 1977 comprehensive review of cough, he described the importance of post-nasal drip as the cause of chronic cough [16]. This was reiterated in a 1981 publication that reported post-nasal drip as the most common cause of chronic cough among 49 consecutive unselected patients with chronic cough. In addition to 29% of the patients having post-nasal drip diagnosed as the cause of cough, cough was due to asthma in 25%, postnasal drip plus asthma in 18%, chronic bronchitis in 12%, gastroesophageal reflux disease (GERD) in 10%, and miscellaneous disorders in 6% [17]. Dr. Irwin and colleagues concluded that the use of an anatomic diagnostic protocol provided that the cause of cough could be consistently determined. This expression of confidence in diagnosing the cause of chronic cough was reiterated in subsequent reviews by Dr. Irwin in 2000 and 2018 [7,18].

That diagnostic certainty was questioned by the 2006 publication of Dr. Rubaiyat Haque and colleagues [19]. They reported that after a thorough, systematic investigation of 100 consecutive unselected patients, 22% had post-nasal symptoms, 16% had GERD, and 7% had asthma as the cause of their chronic cough. But 42% had no identified cause for their chronic cough. Those 42% were considered to be a distinct entity that they called “chronic idiopathic cough.” Two subsequent reviews of chronic cough also reported that over 40% of patients with chronic cough were undiagnosed and were not responsive to usual treatment [20,21]. The terminology for Haque’s chronic idiopathic cough eventually became unexplained and refractory chronic cough [22].

While most of the experience described in the medical literature involved chronic cough in adults, chronic cough also occurs throughout the pediatric spectrum. Unlike in adults, chronic cough in children has rarely been attributed to post-nasal drip or GERD [23]. Etiology of childhood chronic cough relates to age. Protracted bacterial bronchitis, primarily in infants and toddlers, is the most common cause of chronic cough in children [24] but has also been reported in adults [25]. Other reported causes of chronic cough in children include airway malacia involving the trachea and/or bronchi, aspiration including of foreign bodies, asthma, bronchiectasis, cystic fibrosis, post-infectious cough including related to pertussis, and primary ciliary dyskinesia [24,25].

A few case reports of unexplained and refractory chronic cough in children have been published with variable terminology [26,27]. However, it was Dr. Bernard Berman, a pediatric allergist in Boston, who published his experience in 1966 of three boys and three girls, ages 9-13, seen in his practice over five years [15]. They all had a daily involuntary cough for several months that was absent during sleep. In considering terminology for those children, he noted the absence of motor tics, the absence of psychological disorders, and the response to his use of a simple behavioral technique. He stated that he used “the art of suggestion” to provide sustained cessation of cough. Dr. Berman therefore considered the six children to have an involuntary habit disorder. Consistent with the proposal 80 years earlier by Dr. Charles Creighton to “break the habit.” [14], Dr. Berman used suggestion to break the habit cough in all six children. Since then, there have been many published reports regarding childhood habit cough [23,24,25,28]. We are unaware of published documentation of the prevalence of habit cough within the population of all children who present with chronic cough.

## 3. The Present

Chronic cough has been defined as lasting for eight or more weeks. A refractory cough has been defined as persistent despite empirical therapy, regardless of whether a potential physiological cause has been identified [29]. Dr. Richard Irwin, in his 2025 review in the New England Journal of Medicine [22], continued to recommend care for chronic cough by evaluating for asthma, upper airway cough syndrome (formerly post-nasal drip), and GERD as causes of chronic cough. His review restated his prior conclusion [17] that a rigorous systematic review could identify a cause of chronic cough. However, he now acknowledged that 10% could have no identified cause and would be considered unexplained and refractory [22].

In contrast, a recent review in Lancet Respiratory Medicine questioned the reported prevalence of the external causes of chronic cough described by Dr. Irwin. That publication indicated that up to 50% of patients at referral cough clinics had unexplained or refractory chronic cough [30]. In the Rotterdam population-based cohort study, the prevalence of chronic cough was reported by 10.9% of 9824 participants (average age = 66 years) upon their enrollment. Of these, 21% reported unexplained chronic cough, and thus comprised 2.3% of the entire study population. Further, unexplained chronic cough was 40% more likely in patients 70 years or older than in younger adults (2.8% vs. 2.0%). While there was no significant difference related to gender in younger adults, among individuals 70 years and older, women were 25% more likely than men to report an unexplained chronic cough (2.5% vs. 2.0%.) [31]. The Lancet review and the findings from the Rotterdam Study, were consistent with the previous work of Dr. Rubaiyat Haque that chronic cough is a distinct disease rather than a symptom of some disorder. Drs. Woo-Jung Song, Alyn Morice and others proposed cough hypersensitivity as the key explanation for chronic cough. They suggested that various common co-morbidities like GERD and upper airway cough syndrome (UAC) might be triggers, but not the cause [32,33]. This challenged previous paradigms. These authors expressed concern that the assumption of an external cause for chronic cough results in overuse of medications that have little or no effect. Their concept is that chronic cough is the result of neuronal dysregulation involving the brainstem, subcortical, and cortical circuits [34].

Since reflex hypersensitivity was thought to be the key treatable trait, neuromodulation was regarded as needed for cough suppression. However, previous neuromodulators provided little consistent benefit [35]. The first new neuromodulator with more specific receptor effect (a P2X3 receptor antagonist) was gefapixant. Extensive testing of gefapixant, had only a small, though statistically significant effect, on chronic cough and was associated with unpleasant adverse effects [36]. Other P2X3 receptor antagonists such as camlipixant and filapixant have been shown to have somewhat better reduction in cough frequency (up to 37% as compared to placebo) and fewer adverse events in clinical research trials, but to date no P2X3 receptor antagonists have been approved for clinical use by the US Food and Drug Administration [22,37,38]. Opioid receptors present another potential target for pharmaceutical treatment of chronic cough without the potential for abuse such as utilization of dual-acting treatment with kappa receptor agonism and mu receptor antagonism [39].

An alternative explanation for unexplained and refractory chronic cough is based on the observations of local neuropathology caused by the incessant coughing. Inflammation in the airway mucosa was demonstrated in the report of Irwin and colleagues who bronchoscopically obtained mucosal biopsies from young adults with and without chronic cough [40]. The inflammation was the same in those whose cough had no cause as those with an identified cause. Inflammation was not present in volunteers without cough. They suggested that the inflammation in those whose coughing was without a cause occurred because of trauma from the incessant coughing. Perpetuation of cough was also suggested as the cause of increased mucosal nerve density from bronchoscopically obtained samples of otherwise healthy adults with unexplained chronic cough [41]. Chronic cough can therefore be explained as a vicious cycle of mucosal neuropathic inflammation caused by incessant coughing. This is consistent with the clinical awareness of irritation, often described as a “tickle,” that triggers the urge to cough. Hilton and colleagues identified this sensation as the common trait among patients with chronic cough [42].

Suggestion therapy, the behavioral technique described initially by Berman [15], has been used successfully in the treatment of children and adolescents with incessant coughing who report irritation or a “tickle” as a trigger for their cough [43,44]. In one report of suggestion therapy for children, cough cessation was achieved in 95% of 85 patients (average age 10 years) who were coughing at the time suggestion therapy was provided [45]. This method has been used at the University of Iowa Pediatric Allergy and Pulmonary Clinic in hundreds of children diagnosed with habit cough since 1975 (Table 1) [46].

The psychological principles of effective in-person suggestion therapy for refractory cough include:

(1) Helping patients develop positive expectations regarding the therapy. This can be accomplished by explaining the potential physiological and psychological mechanisms that led to the development and persistence of the patients’ symptoms. It is helpful when the clinician is recognized by the patient as being knowledgeable and experienced in taking care of patients with similar conditions [47].
(2) Development of rapport. This can be achieved through inviting patients to fully describe their symptom(s). Ideally, the clinician should be at the eye level of the patients to help the patients feel more empowered. By mirroring the patient’s body posture, rapport can be enhanced. This also helps patients develop a state of focused attention that is helpful for them in accepting suggestions [48].
(3) Teaching the patients to remain patient with their progress, by the modeling of patience and calmness by the clinician [49].
(4) Using verbal and non-verbal language to help convey and maintain positive expectancy while administering the suggestions. For instance, “You’re beginning to feel that you can resist the cough, aren’t you?” that is said with an affirmative nod (Table 1). Such a positive suggestion implying improvement can be much more effective than stating, “Is the feeling that is causing your cough changing?” implying that suggestion therapy can be unhelpful [47].
(5) Acknowledgment of the patients’ progress as the therapy progresses enhances the effectiveness of the suggestions. Further, repetition of the suggestions helps them become better embedded in the mind [50].

These same elements of suggestion therapy for chronic refractory cough also likely underlie the reported:

- 88% improvement but no refractory chronic cough cessation following use of vocal/laryngeal hygiene and cough control techniques in adults who were randomized to a treatment group in a single blinded study (n = 43; average age 58 years) [37,51].
- 90% cough cessation following instruction regarding the use of self-hypnosis in a case series of childhood habit cough (n = 51; average age 11 years) [28].
- 94% cough cessation in a case series of childhood habit cough after the physician convinced pediatric patients that their cough had weakened their chest muscles, which could no longer contain the cough. The physician suggested that a bedsheet tightly wrapped around the chest will provide the necessary support to stop the cough within 24 to 48 h (n = 33; average age 10 years) [52].

Other reported psychological and behavioral therapies for habit cough include use of desensitization techniques, and providing social and monetary rewards for decreasing rates of coughing [53,54].

**Figure 1:**
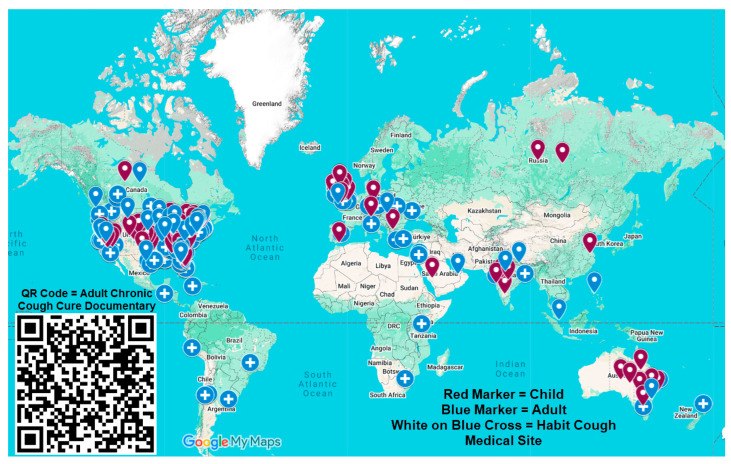
This map provides markers indicating those where the video https://www.youtube.com/watch?v=l6-fffL7Bh0 was viewed and contributed to the cessation of chronic cough. An interactive version of this map is at https://tinyurl.com/HCCureMap (accessed on 11 September 2025). Clicking on a marker of that interactive map provides individual clinical information.

In February 2019, the suggestion therapy method, as described above, for the first time was used remotely by teleconference for a 12-year-old girl with 3 months of refractory chronic cough, which was attributed to habit. A video of that teleconference was published on YouTube and on a website established by the girl’s father (www.habitcough.com (accessed on 11 September 2025)). Many adults and parents of children with chronic intractable cough reported to us by email that viewing the video demonstrating suggestion therapy (https://www.youtube.com/watch?v=l6-fffL7Bh0 11 September 2025) was associated with resolution of their cough or their children’s cough (Figure 1) [46].

Initially, we did not expect that viewing a video of successful suggestion therapy would be effective in treating refractory cough as no rapport could be established by watching a video, nor could viewers receive individualized feedback. In this setting we hypothesize that patients’ responsiveness is attributable to yet another psychological effect, that of identification. For instance, when watching a film, members of an audience can identify with characters in the story and experience their emotions, thoughts, and actions as if they were their own [55]. In this way, people experiencing refractory chronic cough for which they have never found a solution may develop a state of focused attention and respond to suggestions given to another person in a video.

## 4. The Future

Suggestion therapy has become the standard of care for children with refractory chronic cough over the past four years. This was accomplished by dissemination of publications demonstrating a high percentage of cough cessation from suggestion [56,57,58]. Following communication through a pediatric pulmonology list-serve (ped-lung), many pulmonary divisions at academic centers requested presentations to improve their evaluation and care of children with refractory chronic cough. Notably, children with refractory chronic cough have responded to suggestions given during hypnosis, regardless of whether a potential physiological trigger had been identified, such as asthma [28].

Psychosocial stressors have been reported to be associated with occurrence of habit cough in children, such as a recent death, serious physical illness, or mental health crisis of a family member or friend, separation of a child’s parents, or stress related to academic performance such as arising from a learning disability or difficulty living up to the parents’ academic expectations [28]. For some of these children, resolution of cough was facilitated by psychotherapy dealing with its associated psychosocial stressors [28]. Thus, in children, psychotherapy should be offered if suggestion therapy is not associated with rapid cessation of unexplained refractory cough.

Publications demonstrating cessation of refractory chronic cough in adults have been limited and not widely disseminated [56,57]. However, adults who have self-identified cessation of refractory chronic cough after watching and emulating an online video of suggestion therapy provide a proof of concept that suggestion therapy can be an effective means of providing cough cessation in an adult population with a clinical presentation similar to children who were treated successfully for habit cough (Table 2). This may then be the future for helping treat the conundrum of refractory chronic cough in adults.

The goal for the future then is education of physicians who treat adults, especially those likely to be consulted for refractory chronic cough. An understanding of habit cough as a potential disorder in adults is important for physicians to accept this new paradigm. An explanation for the benefit of suggestion can be found in the psychology of suggestion [58] that can influence physical sensations [59]. Chronic refractory daily cough in adults who have a normal exam, spirometry, and chest X-ray is unlikely to be caused by a respiratory disorder. Those with a repetitive, dry daily cough that is absent during sleep may resolve their chronic cough with suggestion therapy.

Since adults who reported cough cessation after viewing a video of successful suggestion therapy were self-selected and not a random sample, the results described in this manuscript cannot be used to assess its potential effectiveness in adults. For instance, no adults reported to us that viewing the suggestion therapy video was unhelpful. However, we have no idea how many adults viewed the video, and what their response might have been. We suspect that adults who may not have contacted us were more likely to have had a poor response to viewing the video. Thus, there is a need to characterize who and how many adults with chronic cough have the clinical characteristics seen in patients who report cough cessation utilizing suggestion therapy. Specifically, who and how many have a repetitive dry cough during most waking hours that is absent during sleep? Those patients could then be evaluated in a controlled clinical trial.

Will the clinical response rate to suggestion therapy depend on the age or gender of adult patients, given the age-related prevalence differences in unexplained chronic cough noted in the Rotterdam study [31]? In children, it is thought that the association of habit cough with psychosocial stressors is consistent with the recognition that psychological factors can be associated with the development, course, or exacerbation of a general medical condition [28,60]. Thus, the increased prevalence of unexplained chronic cough in older and female patients may be related to increased psychosocial stressors in those populations. For instance, Dr. Irwin suggested that women are more likely to develop cough-induced physical symptoms such as stress urinary incontinence, which has psychosocial repercussions [22]. Further, older women are more likely than men to experience common mental disorders such as depression and anxiety [61]. Thus, future studies of unexplained chronic cough should investigate the association of psychosocial stressors with the occurrence of unexplained chronic cough and its response to treatment.

Additionally, research should be undertaken in patients presenting with refractory cough comparing the efficacy and adverse events of neuromodulator therapy with that of suggestion therapy. Available neuromodulators at best reduce the frequency of such cough, while suggestion therapy appears to lead to cessation of refractory chronic cough in children and self-selected adults.

Based on currently available data and our clinical experience, how should those with refractory chronic cough be treated? Since, by definition, these patients have little or no benefit from the usual guideline treatment, it seems to us that the first intervention should be a recommendation that they view the suggestion therapy video, which is free, simple, non-invasive, and does not require administration by a health care professional. Although lacking the financial support of a pharmaceutical company, the epidemiological and psychological investigations for refractory chronic cough may be more fruitful than current efforts to find the perfect neuromodulator for treatment of cough hypersensitivity.

## Figures and Tables

**Table 1:** Major elements of suggestion therapy.

Approach the patient with confidence that the coughing will be stopped. Explain the cough as a vicious cycle that started with an initial irritant that is now gone and that now the cough itself is causing irritation and more cough. Instruct the patient to concentrate solely on holding back the urge to cough for an initially brief timed period, such as 1 min. Progressively increase this time period and use an alternative behavior, such as sipping lukewarm water or inhaling a soothing cool mist from a vaporizer, to “ease the irritation.” Tell the patient that each second the cough is delayed makes it easier to suppress further coughing. Repeat expressions of confidence that the patient is developing the ability to resist the urge to cough: “It’s becoming easier to hold back the cough, isn’t it?” (Nodding affirmatively generally results in a similar affirmation movement by the patient.) When ability to suppress cough is observed (usually by about 10 min), ask in a rhetorical manner, “You’re beginning to feel that you can resist the urge to cough, aren’t you?” (said with an affirmative head nod) Discontinue the session when the patient can repeatedly respond positively to the question, “Do you feel that you can now resist the urge to cough on your own?” This question is only asked after the patient has gone 5 min without coughing. Express confidence that if the urge to cough recurs that the patient can do the same thing at home (autosuggestion). *

* Autosuggestion involved expressing confidence in 15 min sessions at home concentrating on holding back the cough with sips of lukewarm water to “ease the irritation causing cough.” [46].

**Table 2:** 42 adults who self-identified cessation of chronic cough (as of March, 2025) after watching an online video of suggestion therapy (https://youtu.be/jnQUvD8Qdj0 (accessed on 11 September 2025)). “**r**” identifies those where viral respiratory infections (VRI) repeatedly resulted in many months of chronic cough with eventual spontaneous resolution and recurrence following the next VRI.

Contact Date	Sex	Age (yr)	Location	Cough Duration (Months)
3/23/2019	F	68	Canada	72
6/9/2019	F	58	Minnesota	12
6/25/2019	M	22	Iowa	24
9/5/2019	M	27	Canada	36
12/13/2019	M	26	Nepal	60
1/15/2020	M	60	Philippines	180
7/14/2020	F	24	Singapore	9
8/4/2020	F	62	Iowa	120
9/2/2020	F	41	Florida	60
9/16/2020	M	23	Illinois	120
9/21/2020	F	41	Nebraska	60
10/17/2020	M	21	India	5
10/28/2020	F	44	California	17
10/30/2020	F	41	Ireland	20
11/4/2020	M	30	Minnesota	24
11/28/2020	M	25	Florida	4
12/1/2020	M	53	Texas	240
7/31/2021	M	42	Illinois	30
9/21/2021	F	24	Canada	168
12/2/2021	M	29	Hungary	4
12/12/2021	F	38	New Jersy	**r**60
5/22/2022	F	61	Maryland	180
8/7/2022	F	31	New York	**r**120
8/29/2022	F	70	California	66
10/4/2022	M	22	Vietnam	8
3/15/2023	M	42	New Hampshire	**r**240
4/17/2023	F	34	Michigan	17
9/29/2023	F	45	Indiana	36
10/22/2023	M	53	Virginia	**r**240
1/5/2024	F	58	Ohio	144
4/2/2024	M	44	New Jersey	**r**168
5/3/2024	F	36	Indonesia	**r**228
5/24/2024	F	70	Michigan	24
5/28/2024	F	67	Canada	40
6/13/2024	F	49	England	18
10/4/2024	M	57	St. Louis	192
10/14/2024	F	41	Utah	2
10/25/2024	F	48	Florida	3
12/27/2024	M	34	Oregon	300
1/9/2025	M	24	India	36
1/19/2025	M	34	England	48
3/3/2025	F	42	New York	60

## Data Availability

Data in the form of parent or patient email communications are available for purposes of further research of habit cough.

## References

[1] Doan T., Patterson R., Greenberger P.A. (1992). Cough variant asthma: Usefulness of a diagnostic-therapeutic trial with prednisone. Ann. Allergy.

[2] Sykes D.L., Mason P., Rahunathan N., Hart S.P., Morice A.H., Crooks M.G. (2024). The Effect of Long-Term Azithromycin on Objective and Subjective Cough in Chronic Respiratory Disease: A Systematic Review and Meta-analysis of Randomised Controlled Trials and Noncomparative Studies. Lung.

[3] Myall K.J., Cho P.S.P., Birring S.S. (2024). What causes cough in pulmonary fibrosis, and how should we treat it?. Curr. Opin. Pulm. Med..

[4] Semple P.F., Herd G.W. (1986). Cough and wheeze caused by inhibitors of angiotensin-converting enzyme. N. Engl. J. Med..

[5] Gurgel R.K., Brookes J.T., Weinberger M.M., Smith R.J. (2008). Chronic cough and tonsillar hypertrophy: A case series. Pediatr. Pulmonol..

[6] Song W.J., Chang Y.S., Faruqi S., Kim J.Y., Kang M.G., Kim S., Jo E.J., Kim M.H., Plevkova J., Park H.W. (2015). The global epidemiology of chronic cough in adults: A systematic review and meta-analysis. Eur. Respir. J..

[7] Irwin R.S., French C.L., Chang A.B., Altman K.W., CHEST Expert Cough Panel* (2018). Classification of Cough as a Symptom in Adults and Management Algorithms: CHEST Guideline and Expert Panel Report. Chest.

[8] Morice A.H., Millqvist E., Bieksiene K., Birring S.S., Dicpinigaitis P., Domingo Ribas C., Hilton Boon M., Kantar A., Lai K., McGarvey L. (2020). ERS guidelines on the diagnosis and treatment of chronic cough in adults and children. Eur. Respir. J..

[9] Prentice D.A. (2024). Cough in Children and Adults: Diagnosis, Assessment and Management (CICADA). Summary of an updated position statement on chronic cough in Australia. Med. J. Aust..

[10] Rouadi P.W., Idriss S.A., Bousquet J., Morais-Almeida M., Azar C.R., Al-Ahmad M.S., Yáñez A., Ali YAl-Nesf M., Nsouli T.M., Bahna S.L. (2025). WAO—ARIA consensus on chronic cough: Executive summary. World Allergy Organ. J..

[11] Willis T. (1685). The London Practice of Physick, in the Pharmaceutic Rationalis.

[12] Mercurius F. (1694). Habitual Cough. The Spirit of Diseases.

[13] Weinberger M. (2018). The habit cough: Diagnosis and treatment. Pediatr. Pulmonol..

[14] Creighton C. (1886). Illustrations of Unconscious Memory in Disease.

[15] Berman B.A. (1966). Habit cough in adolescent children. Ann. Allergy.

[16] Irwin R.S., Rosen M.J., Braman S.S. (1977). Cough: A comprehensive review. Arch. Intern. Med..

[17] Irwin R.S., Corrao W.M., Pratter M.R. (1981). Chronic persistent cough in the adult: The spectrum and frequency of causes and successful outcome of specific therapy. Am. Rev. Respir. Dis..

[18] Irwin R.S., Madison J.M. (2000). The diagnosis and treatment of cough. N. Engl. J. Med..

[19] Haque R.A., Usmani O.S., Barnes P.J. (2005). Chronic idiopathic cough: A discrete clinical entity?. Chest.

[20] Smith J.A., Woodcock A. (2016). Chronic Cough. N. Engl. J. Med..

[21] Gibson P.G. (2019). Management of Cough. J. Allergy Clin. Immunol. Pract..

[22] Irwin R.S., Madison J.M. (2025). Unexplained or Refractory Chronic Cough in Adults. N. Engl. J. Med..

[23] Chang A.B., Oppenheimer J.J., Weinberger M., Weir K., Rubin B.K., Irwin R.S. (2016). Use of Management Pathways or Algorithms in Children with Chronic Cough: Systematic Reviews. Chest.

[24] Chang A.B., Robertson C.F., Van Asperen P.P., Glasgow N.J., Mellis C.M., Masters I.B., Teoh L., Tjhung I., Morris P.S., Petsky H.L. (2012). A multicenter study on chronic cough in children: Burden and etiologies based on a standardized management pathway. Chest.

[25] Weinberger M., Hurvitz M. (2020). Diagnosis and management of chronic cough: Similarities and differences between children and adults. F1000Research.

[26] Bernstein L. (1963). A respiratory tic: “The barking cough of puberty.” Report of a case treated successfully. Laryngoscope.

[27] Baker D.C. (1963). Chronic cough in children. N. Y. State J. Med..

[28] Anbar R.D., Hall H.R. (2004). Childhood habit cough treated with self-hypnosis. J. Pediatr..

[29] Gibson P., Wang G., McGarvey L., Vertigan A.E., Altman K.W., Birring S.S., CHEST Expert Cough Panel (2016). Treatment of Unexplained Chronic Cough: CHEST Guideline and Expert Panel Report. Chest.

[30] Chung K.F., Mazzone S.B., McGarvey L., Song W.J. (2025). Chronic cough as a disease: Implications for practice, research, and health care. Lancet Respir. Med..

[31] Arinze J.T., van der Veer T., Bos D., Stricker B., Verhamme K.M.C., Brusselle G. (2023). Epidemiology of unexplained chronic cough in adults: A population-based study. ERJ Open Res..

[32] Song W.J., Chang Y.S., Morice A.H. (2014). Changing the paradigm for cough: Does ‘cough hypersensitivity’ aid our understanding?. Asia Pac. Allergy.

[33] Song W.J., Morice A.H. (2017). Cough Hypersensitivity Syndrome: A Few More Steps Forward. Allergy Asthma Immunol. Res..

[34] Song W.J., Manian D.V., Kim Y., Zhang M., Morice A.H. (2025). Cough Reflex Hypersensitivity as a Key Treatable Trait. J. Allergy Clin. Immunol. Pract..

[35] Sandham K., Emmett S., Nguyen D.D., Madill C., Novakovic D. (2025). Predictive Factors and Treatment Effects of Neuromodulators in Chronic Refractory Cough. J. Otolaryngol. Head. Neck Surg..

[36] McGarvey L.P., Birring S.S., Morice A.H., Dicpinigaitis P.V., Pavord I.D., Schelfhout J., Nguyen A.M., Li Q., Tzontcheva A., Iskold B. (2022). Efficacy and safety of gefapixant, a P2X_3_ receptor antagonist, in refractory chronic cough and unexplained chronic cough (COUGH-1 and COUGH-2): Results from two double-blind, randomised, parallel-group, placebo-controlled, phase 3 trials. Lancet.

[37] Slovarp L.J., Reynolds J.E., Gillespie A.I., Jetté M.E. (2025). Reframing Refractory Chronic Cough: The Role of Interoception. Lung.

[38] Smith J.A., Birring S.S., Blaiss M.S., McGarvey L., Morice A.H., Sher M., Carroll K.J., Garin M., Lanouette S., Shaw J. (2025). Camlipixant in Refractory Chronic Cough: A Phase 2b, Randomized, Placebo-controlled Trial (SOOTHE). Am. J. Respir. Crit. Care Med..

[39] Birring S.S., Dicpinigaitis P.V., Maher T.M., Mazzone S.B., Page C.P., Hawi A., Sciascia T., Morice A.H. (2025). Kappa and Mu Opioid Receptors in Chronic Cough: Current Evidence and Future Treatment. Lung.

[40] Irwin R.S., Ownbey R., Cagle P.T., Baker S., Fraire A.E. (2006). Interpreting the histopathology of chronic cough: A prospective, controlled, comparative study. Chest.

[41] Shapiro C.O., Proskocil B.J., Oppegard L.J., Blum E.D., Kappel N.L., Chang C.H., Fryer A.D., Jacoby D.B., Costello R.W., Drake M.G. (2021). Airway Sensory Nerve Density Is Increased in Chronic Cough. Am. J. Respir. Crit. Care Med..

[42] Hilton E., Marsden P., Thurston A., Kennedy S., Decalmer S., Smith J.A. (2015). Clinical features of the urge-to-cough in patients with chronic cough. Respir. Med..

[43] Kim Y., Birring S.S., McGarvey L., Morice A.H., Song W.J. (2025). How Will a Treatable Traits Approach Reshape Clinical Practice in Chronic Cough?. Allergy Asthma Immunol. Res..

[44] Weinberger M., Buettner D. (2024). The Habit Cough Syndrome. Pediatr. Pulmonol..

[45] Weinberger M., Lockshin B. (2017). When is cough functional, and how should it be treated?. Breathe.

[46] Weinberger M., Hoegger M. (2016). The cough without a cause: Habit cough syndrome. J. Allergy Clin. Immunol..

[47] Kube T., Glombiewski J.A., Rief W. (2018). Using different expectation mechanisms to optimize treatment of patients with medical conditions: A systematic review. Psychosom. Med..

[48] Leach M.J. (2005). Rapport: A key to treatment success. Complement. Ther. Clin. Pract..

[49] Hashim M.J. (2017). Patient-Centered Communication: Basic Skills. Am. Fam. Physician..

[50] Oakley D.A., Halligan P.W. (2009). Hypnotic suggestion and cognitive neuroscience. Trends Cogn. Sci..

[51] Vertigan A.E., Theodoros D.G., Gibson P.G., Winkworth A.L. (2006). Efficacy of speech pathology management for chronic cough: A randomised placebo controlled trial of treatment efficacy. Thorax.

[52] Cohlan S.Q., Stone S.M. (1984). The cough and the bedsheet. Pediatrics.

[53] Lavigne J.V., Davis A.T., Fauber R. (1991). Behavioral management of psychogenic cough: Alternative to the “bedsheet” and other aversive techniques. Pediatrics..

[54] Fulcher R., Cellucci T. (1997). Case formulation and behavioral treatment of chronic cough. J. Behav. Ther. Exp. Psychiatry.

[55] Igartua J.J. (2010). Identification with characters and narrative persuasion through fictional feature films. Communications.

[56] Weinberger M., Buettner D. (2023). Habit cough is a cause of chronic cough in adults. Ann. Allergy Asthma Immunol..

[57] Weinberger M., Anbar R.D., Buettner D. (2025). Interrupt a vicious cycle to Cure refractory chronic cough. Am. J. Otolaryngol..

[58] Michael R.B., Garry M., Kirsch I. (2012). Suggestion, Cognition, and behavior. Curr. Dir. Psychol. Sci..

[59] Peerdeman K.J., van Laarhoven A.I., Donders A.R., Hopman M.T., Peters M.L., Evers A.W. (2015). Inducing Expectations for Health: Effects of Verbal Suggestion and Imagery on Pain, Itch, and Fatigue as Indicators of Physical Sensitivity. PLoS ONE.

[60] Martino G., Langher V., Cazzato V., Vicario C.M. (2019). Editorial: Psychological Factors as Determinants of Medical Conditions. Front. Psychol..

[61] Kiely K.M., Brady B., Byles J. (2019). Gender, mental health and ageing. Maturitas.

